# An Experimental Demonstration of the Use of Pepto-Mangan “Gude”1Read before the Memphis Medical Society.

**Published:** 1906-07

**Authors:** William Krauss

**Affiliations:** Memphis, Tenn., Director of the Microscopic Laboratories, Memphis Medical College; Pathologist and Visiting Physician to St. Joseph’s Hospital


### SELECTIONS.

### An Experimental Demonstration of the Use of Pepto-Mangan "Gude."[1]

By WILLIAM KRAUSS, Ph. G., M. D., Memphis, Tenn.,
Director of the Microscopic Laboratories, Memphis Medical College; Pathologist and Visiting Physician to St. Joseph's Hospital

(*Memphis Lancet, April, 1900.*)

SOME five years ago I wrote a paper for the *Memphis Medical Monthly,* giving a résumé of the evolution of the iron compounds, and appended a report of cases giving blood counts, etc. The manufacturers of the preparation I preferred, saw fit to reproduce the case reports in their pamphlets, but said nothing about the reasons that induced me to prefer their product.

1. Read before the Memphis Medical Society.

At a recent joint meeting of physicians and pharmacists I was criticised for opposing the use of ready-made compounds, while still advocating the use of Pepto-Mangan "Gude," which is a proprietary preparation. I hesitated considerably about bringing the matter up again, because I dislike to build up a reputation as an endorser, and have never in any other instance written an article endorsing a proprietary preparation.

I hope, however, to show you this evening that there is no pharmacopeial preparation that meets the requirements of an ideal iron compound, and, until this is found, I intend to continue to use what has never disappointed me and is not based upon mere faith. The work of Bunge is too well known to be now quoted, and I will only make a few experiments before you this evening and show the reasons for the faith that is in me. There may be other proprietary iron compounds, and doubtless there are, that will come up to the same requirements, but I see no advantage in swapping the devil for the witch. It is not necessary to repeat all the tests with all the official iron preparations, because they are divisible into groups, all the salts of one group behaving very much alike toward the gastric and intestinal juices.

An ingenious theory recently put forward regarding the action of the mineral salts of iron is, that they decompose the substances in the intestinal tract which precipitate the food iron so that it may be absorbed. This is the only rational explanation of the fact that we do occasionally get results from them. On the other hand, it is far more rational to use an iron compound that can be, and is absorbed, for then we are reckoning with known quantities, instead of blundering along, giving more iron at a dose than is contained in the entire body, and incidentally deranging the digestive functions by precipitating the gastric, pancreatic and intestinal juices, and producing constipation by reason of the very astringent nature of some of the iron salts. Beginning with the organic double salts, of which the scale salts are representatives, we notice upon the addition of this gastric juice, that a precipitate is formed; the double salt is decomposed and ferric salt remains, which is insoluble, both in gastric and intestinal juice.

The tincture of ferric chloride will precipitate some of the gastric constituents, though most of the iron will remain in solution in the hydrochloric acid; the iron still in solution will not be absorbed, because its non-diffusibility is taken advantage of in the manufacture of dialyzed iron, the acid passing through the animal membrane; when the iron finally reaches the intestine, the alkaline carbonates promptly precipitate it. Ferrous sulphate behaves similarly. In both instances, as you see, the very insoluble ferric oxide is finally formed. If you have ever tried to remove iron stains from your water pitcher, you have some idea how insoluble it is. The insoluble compounds, like reduced iron, or Vallet's mass, only serve to render inert the arsenic with which they are usually prescribed; if dissolved at all in the stomach, they are re-precipitated in the intestine.

Taking now Gude's preparation, we find it soluble, not only in all these reagents, but also in a mixture of them. Potassium ferrocyanide readily gives the iron reaction, excess of ammonia will separate it, redissolving the manganese, which is then recognised by the color of its sulphide; the alkaline copper solution gives the reaction for peptone, showing that it is what the label says. It mixes with arsenious acid, forming a perfect solution, thus giving us a most useful hematopoietic agent. The soluble alkaloids are perfectly soluble in it, as is also mercuric chloride. Being a peptone, it is readly diffusible by osmosis. The only disturbing agent in the intestinal tract is hydrogen sulphide; this will precipitate it, but presumably, much of the iron must have been absorbed before it encounters this gas; if not, appropriate agents should be used for its elimination. Therapeutically, it does not nauseate, constipate, discolor the teeth, precipitate the digestive agents nor become inert from contact with them. As to the clinical results, I need not add anything to the many reports already on record.

